# Intradermally Administered Retinoic Acid or Vitamin D3-Loaded Liposomes Induce Tolerogenic Skin Dendritic Cells

**DOI:** 10.1155/jimr/2208155

**Published:** 2025-08-04

**Authors:** Noémi Anna Nagy, Sanne G. Celant, Toni M. M. van Capel, Rinske Sparrius, Fernando Lozano Vigario, Ronald van Ree, Bram Slütter, Teunis B. H. Geijtenbeek, Sander W. Tas, Esther C. de Jong

**Affiliations:** ^1^Department of Experimental Immunology, Amsterdam University Medical Centers, Amsterdam Institute for Infection and Immunity, Amsterdam, Netherlands; ^2^Division of BioTherapeutics, Leiden Academic Center for Drug Research, Leiden University, Leiden, Netherlands; ^3^Department of Otorhinolaryngology, Amsterdam University Medical Centers, Amsterdam, Netherlands; ^4^Department of Rheumatology and Clinical Immunology, Amsterdam University Medical Centers, Amsterdam Rheumatology and Immunology Center, Amsterdam, Netherlands

## Abstract

In vivo targeting of dendritic cells (DCs) with nanocarriers containing tolerogenic adjuvants is an attractive strategy to dampen inflammation. Here, we used ex vivo skin vaccination to examine the effect of intradermal injection of liposomes loaded with the tolerogenic adjuvants all-trans retinoic acid (RA) and vitamin D3 (VD3). We investigated the effect of intradermal liposome injection on skin DCs and the skin DC-induced T cell response. Our study shows that intradermal injection of RA or VD3-loaded anionic phospholipid 1,2-distearoyl-sn-glycero-3-phosphoglycerol (DSPG) liposomes selectively induces CD14+ dermal DC (DDC) migration while reducing migration of CD1a dim DDCs. Migrated CD14+ DDCs displayed a partially immature phenotype. RA or VD3 liposome-treated CD1a dim DDCs exhibited reduced expression of maturation markers and induced expression of coinhibitory immunoglobulin-like transcript 3 (ILT3). VD3 liposome-treated CD14+ DDCs, as well as, CD1a dim DDCs, exhibited reduced expression of maturation markers, induction of coinhibitory molecules ILT3, and programmed death-ligand 1 (PD-L1). Migrated DCs from RA or VD3 liposome-injected skin differentiated naïve CD4+ T cells into FoxP3+ CD127 low and ICOS+ Tregs, expressing functional regulatory markers. Thus, our findings provide further substantiation for in vivo DC-modulating vaccines with tolerogenic liposomes as a putative clinical therapy for autoimmune diseases and allergies.

## 1. Introduction

Autoimmune disorders and allergies may result from loss of immune tolerance [1]. Dendritic cells (DCs) are critical antigen presenting cells (APCs) that guide immunity towards either inflammation or tolerance. This duality makes DCs highly suitable for treatment of cancer, autoimmune diseases, or allergic conditions [2–4]. DCs residing in tissues, such as the lung, intestine, and skin may induce both regulatory T and B cells by the secretion of suppressive cytokines or metabolites or expression of inhibitory molecules with tolerogenic activity [5, 6].

Various clinical studies use ex vivo DC vaccination as a strategy to achieve tolerance and suppress immunity [7–9]. These trials have shown that tolerogenic DC therapy is feasible and effective for the treatment of several autoimmune diseases and organ transplants. However, harvesting DCs or their precursors from patients and differentiating these cells in vitro is an expensive and difficult process, often accompanied by undesired functionality of DCs. In contrast, in vivo targeting of DCs has significant potential as DCs are modulated within their natural niche.

It is long recognized that all-trans retinoic acid (RA) and 1α,25-dihydroxyvitamin D3, the active form of vitamin D3 (VD3), promote the induction of tolerogenic properties in DCs [10–15]. RA can also work as a stimulator of immune cell proliferation and expansion when immune reconstitution is needed [16, 17]. However, particularly in mucosal immunity, but also towards commensals in the skin, RA has been described as tolerance-promoting [14, 18]. VD3, on the other hand, is unanimously reported as strongly tolerogenic. VD3 administered together with autoantigen leads to DC-driven antigen-specific memory T cell anergy and antigen-specific Treg induction [19]. Of note, topically administered analogues of RA and VD3 are already in use as treatment for inflammatory skin diseases, such as psoriasis vulgaris, illustrating the potential clinical application of RA- and VD3-based approaches [20, 21]. Hence, using RA and VD3 in a dermal delivery context could be a putative treatment for various inflammatory disorders. The skin is easily accessible for injection and harbors a network of heterogenous populations of DCs, readily available for in vivo immune modulation. DC subsets in the skin include epidermal Langerhans cells (LCs) and a variety of DC subsets populating the dermis, with CD14+ and CD1a dim dermal DCs (DDCs) forming two major DDC subsets [22–24].

One approach to target DCs in vivo for immune modulation is using DC-targeted nanocarriers, such as liposomes, to deliver vaccine compounds, such as immune tolerogenic adjuvants, solely or combined with disease-associated antigens [25–27]. Liposomes are nanoparticles consisting of a phospholipid bilayer and allow for simple modification of various characteristics, including size, rigidity, and electric charge. Furthermore, several types of liposomes already have FDA approval [28]. Liposomes of varying lipid compositions, and electric surface charge can be used to target APCs and modulate their function. For instance, anionic 1,2-distearoyl-sn-glycero-3-phosphoglycerol (DSPG) liposomes have been shown to induce Tregs in mice. Moreover, DSPG liposomes were more readily internalized by human monocyte-derived DCs (moDCs) than cationic formulations with similar lipid composition [29–31]. However, little is known about targeting skin DCs with RA or VD3 liposomes and whether these formulations induce immune tolerance.

Here, we demonstrate that intradermal injection of RA- or VD3-loaded DSPG liposomes selectively increases CD14+ DDC migration. Moreover, RA and VD3-stimulated DCs migrating from skin prime naïve CD4+ T cells to differentiate into FoxP3+ T cells and ICOS+ Tregs expressing functional Treg markers, possibly through reduced activation markers and expression of tolerogenic molecules. These findings provide proof-of-concept for an in vivo DC modulating vaccine using tolerogenic liposomes as a putative clinical therapy for autoimmune diseases and allergies.

## 2. Results

### 2.1. RA- and VD3-Loaded DSPG Liposomes Induce Migration of CD14+ DDCs From Human Skin

**Figure 1:**
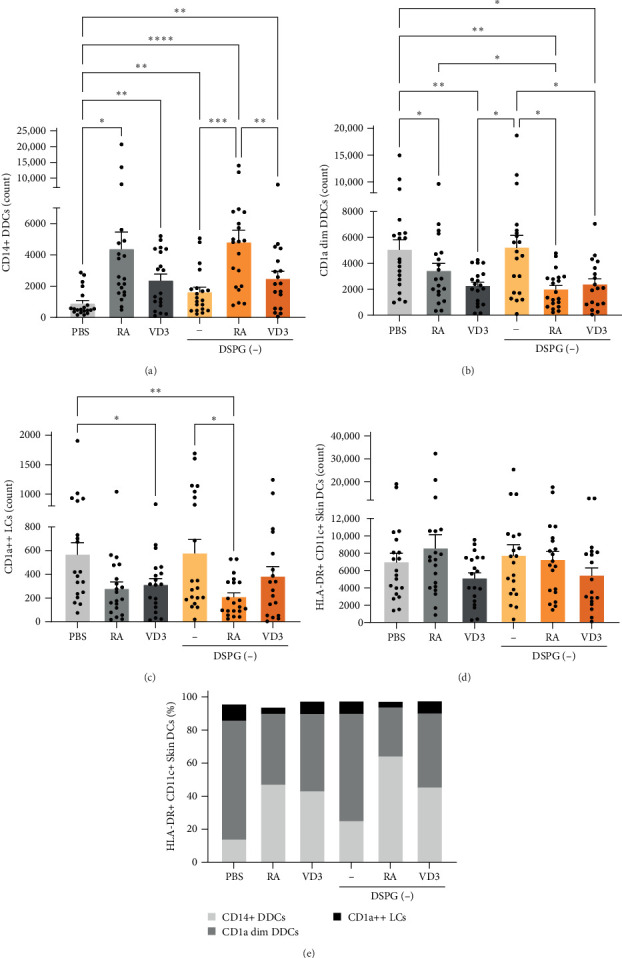
Ex vivo RA and VD3 liposome injection enhances migration of CD14+ DDCs while reducing migration of CD1a dim DDCs and CD1a++ LCs. 30 μM RA or 25 μM VD3 was injected in human skin in soluble form or loaded in anionic DSPG liposomes. 6 mm biopsies taken from the injection site were cultured for 3 days and crawl-outs migrating from the biopsies harvested for phenotypic analysis. Surface expression of skin DC markers and skin DC subset markers was analyzed using spectral flow cytometry. (A–C) Counts of CD14+ DDCs, CD1a dim DDCs, and CD1a++ LCs that migrated from skin explants within HLA-DR+ CD11c+ skin DCs. (D) Counts of HLA-DR+ CD11c+ skin DCs that migrated from skin explants. (E) Each skin DC subset's frequency is shown per injection condition within the HLA-DR+ CD11c+ population of crawl-out DCs. *N* = 18–20. Error bars indicate mean ± SEM. Statistical significance was calculated using mixed-effects analysis with Tukey's correction for multiple comparisons. *⁣*^*∗*^*p* ≤ 0.05. *⁣*^*∗∗*^*p* ≤ 0.01. *⁣*^*∗∗∗*^*p* ≤ 0.001. *⁣*^*∗∗∗∗*^*p* ≤ 0.0001. Individual data points: number of donors tested per condition (independent experiments).

Adjuvants can influence the composition of migratory skin DC subests. We have previously demonstrated that injection of soluble or DSPG liposome-loaded VD3 led to increased CD14+ DDC migration from human skin explants [32, 33]. To investigate the effect of intradermal administration of RA and DSPG liposomes containing RA on the migratory patterns of skin DCs, we determined the absolute cell number (count) of DCs crawling out from injected skin biopsies by flow cytometry (Figure S1A). Skin DCs were identified by high expression levels of both HLA-DR and CD11c (Figure S1A), and gated further based on their CD1a and CD14 expression, distinguishing CD14+ DDCs, CD1a dim DDCs and CD1a++ LCs. Of note, 61.8%–98.6% of CD14+ DDCs coexpressed CD141, suggesting an alignment with conventional DCs type 1 (CDC1), whereas only 9.5%–24.6% of CD1a dim DDCs expressed CD141, indicating that the majority of these cells aligns with a different cDC subset (Figure S1B,C). Injection of liposomes or soluble compounds did not affect cell viability, nor frequencies of HLA-DR+ CD11c+ skin DCs (Figure S1D,E). Injection of liposomes with RA significantly enhanced CD14+ DDC migration, compared to PBS and empty DSPG injection (Figure 1A, Supporting Information 2: Figure S2A). In contrast, CD1a dim DDC and CD1a++ LC migration was inhibited by RA and RA liposome injection (Figure 1B,C, Supporting Information 2: Figure S2A). Interestingly, empty DSPG liposomes also induced CD14+ DDC migration compared to PBS, while not affecting CD1a dim DDC nor LC migration (Figure 1A–C). Counts of total HLA-DR+ CD11c+ skin DCs did not differ significantly between injection conditions (Figure 1D). Consequently, relative frequencies of each skin DC population followed a similar trend as number of migrating DC subsets (Figure 1E, Supporting Information 2: Figure S2B–D). Hence, RA liposome injection increased frequencies of migrating CD14+ DDCs and decreased frequencies of both CD1a dim DDCs and CD1a++ LCs (Figure 1E, Supporting Information 2: Figure S2B–D). As previously published, soluble or DSPG liposome-loaded VD3 injection increased migration of CD14+ DDCs from ex vivo skin (Figure 1A,Figure 1E,Supporting Information 2: Figure S2A–D) [32, 33]. Thus, our data suggest that soluble RA or RA liposomes, as well as, VD3 liposomes enhance migration of CD14+ DDCs from skin, while inhibiting migration of CD1a dim DDC and LCs.

### 2.2. RA and VD3-Liposome Injection Suppress DC Activation and Induce Tolerogenic Markers of Skin DC Subsets

To determine the influence of intradermally injected RA and VD3 liposomes on the phenotype of skin DCs, we measured the expression of the DC activation markers CD40, CD83, CD86, and the tolerogenic markers B7H3, ICOS-L, ILT2, ILT3, and PD-L1 within the different DC populations by spectral flow cytometry (Figure S1A).

**Figure 2:**
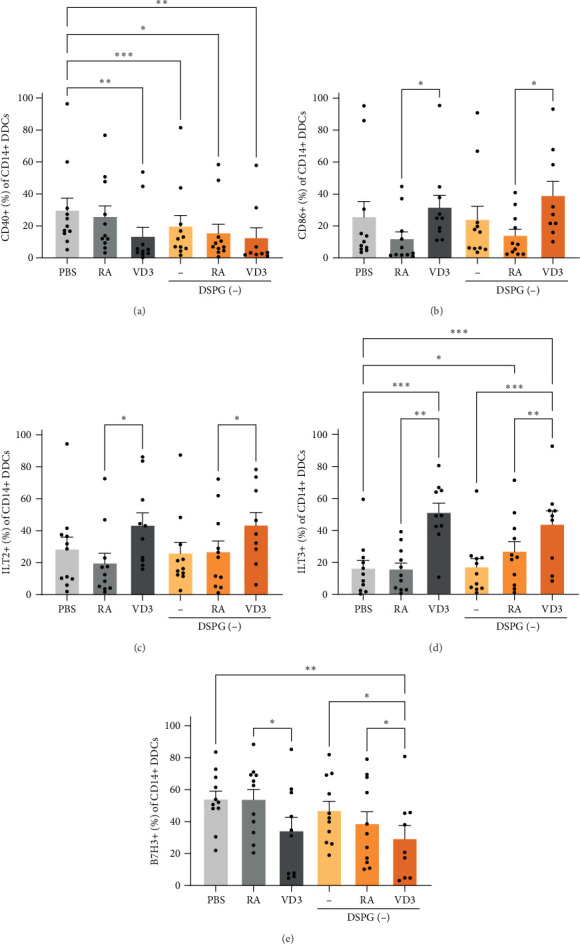
RA and VD3 liposome treatment modulate expression of skin DC-activating and tolerogenic markers in less mature CD14+ DDCs. Frequencies of marker+ cells were evaluated in the different injection conditions within HLA-DR+ CD11c+ skin DCs, which were CD14+, using spectral flow cytometry. Conditions with counts less than 100 CD14+ DDCs were excluded from analysis. (A,B) Frequencies of CD40+, and CD86+ CD14+ DDCs are shown. (C–E) Frequencies of CD14+ DDCs expressing ILT2, ILT3, and B7H3 are shown. *N* = 9–11. Error bars indicate mean ± SEM. Statistical significance was calculated using mixed-effects analysis with Tukey's correction for multiple comparisons. *⁣*^*∗*^*p* ≤ 0.05. *⁣*^*∗∗*^*p* ≤ 0.01. *⁣*^*∗∗∗*^*p* ≤ 0.001. Individual data points: number of donors tested per condition (independent experiments).

RA alone or in liposomes did not significantly modulate CD14+ DDC marker expression (Figure 2A–E). Interestingly, VD3 alone or in liposomes reduced the frequencies of CD40+ and CD14+ DDCs while inducing expression of coinhibitory markers immunoglobulin-like transcript (ILT2) and ILT3 on CD14+ DDCs compared to PBS injection, RA, or empty liposomes (Figure 2A,C,D). Both VD3 and VD3 liposomes induced CD86 expression compared to RA, while reducing coinhibitory B7H3+ CD14+ DDCs (Figure 2B,E). Similar results were obtained when measuring MFI rather than percentages (Figure S3A–E). These data suggest that VD3 but not RA induces a partial tolerogenic phenotype in CD14+ DDCs.

**Figure 3:**
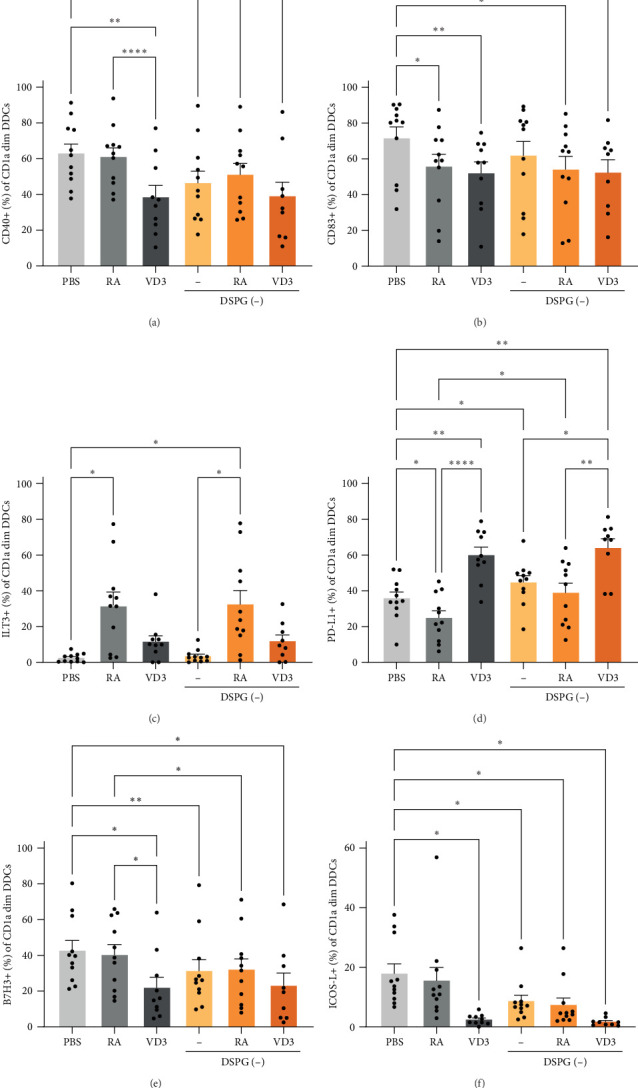
RA and VD3 liposome injection reduce expression of activation markers and induce expression of tolerogenic markers in mature CD1a dim DDCs. Frequencies of marker+ cells were evaluated in the different injection conditions within HLA-DR+ CD11c+ skin DCs, which were CD1a dim, using spectral flow cytometry. Conditions with counts less than 100 CD1a dim DDCs were excluded from analysis. (A,B) Frequencies of CD40+, and CD83+ CD1a dim DDCs are shown. (C–F) Frequencies of CD1a dim DDCs expressing ILT3, PD-L1, B7H3, and ICOS-L are shown. *N* = 9–11. Error bars indicate mean ± SEM. Statistical significance was calculated using mixed-effects analysis with Tukey's correction for multiple comparisons. *⁣*^*∗*^*p* ≤ 0.05. *⁣*^*∗∗*^*p* ≤ 0.01. *⁣*^*∗∗∗∗*^*p* ≤ 0.0001. Individual data points: number of donors tested per condition (independent experiments).

Within CD1a dim DDCs both liposome-loaded RA and VD3 reduced CD40 and CD83 expression, comparably to soluble vitamin controls (Figure 3A,B, Supporting Information 4: Figure S4A,B). In this DC subset ILT3 expression was only induced by RA as well as RA liposomes, while VD3 and VD3 DSPG injection induced PD-L1+ CD1a dim DDCs and PD-L1 MFI (Figure 3C,D, Supporting Information 4: Figure S4C,D). Interestingly, VD3 treatment also reduced coinhibitory B7H3+ and ICOS-L+ CD1a dim DDCs, as well as MFI, while ICOS-L expression was not altered in the other subsets (Figure 3E,F, Supporting Information 4: Figure S4E,F).

**Figure 4:**
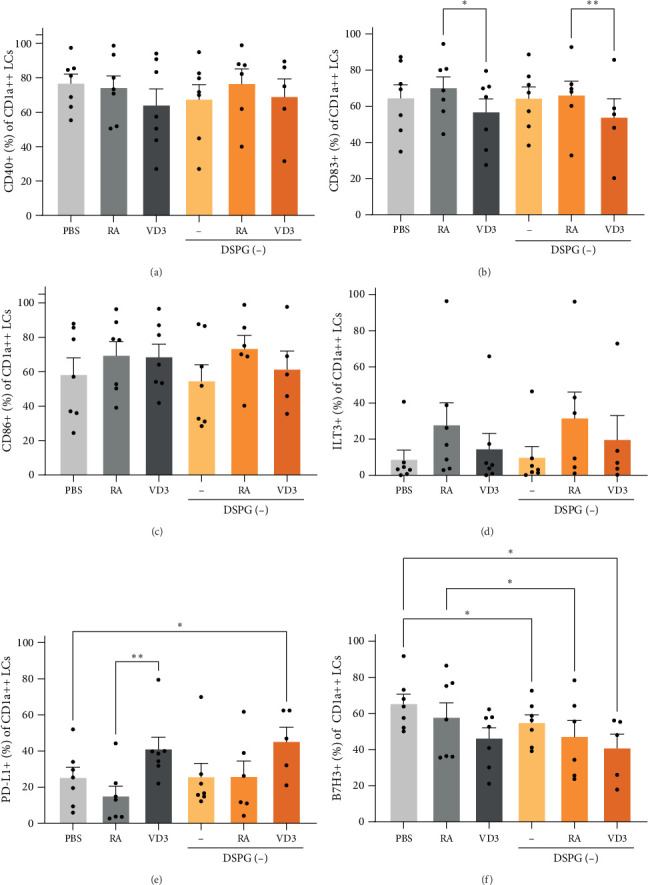
RA and VD3 liposome injection do not alter expression of activation markers on LCs but modulate tolerogenic marker expression. Frequencies of marker+ cells were evaluated in the different injection conditions within HLA-DR+ CD11c+ skin DCs, which were CD1a++, using spectral flow cytometry. Conditions with counts less than 100 CD1a++ LCs were excluded from analysis. (A–C) Frequencies of CD40+, CD83+, and CD86+ CD1a++ LCs are shown. (D–F) Frequencies of CD1a++ LCs expressing ILT3, PD-L1, and B7H3 are shown. *N* = 5–7. Error bars indicate mean ± SEM. Statistical significance was calculated using mixed-effects analysis with Tukey's correction for multiple comparisons. *⁣*^*∗*^*p* ≤ 0.05. *⁣*^*∗∗*^*p* ≤ 0.01. Individual data points: number of donors tested per condition (independent experiments).

**Figure 5:**
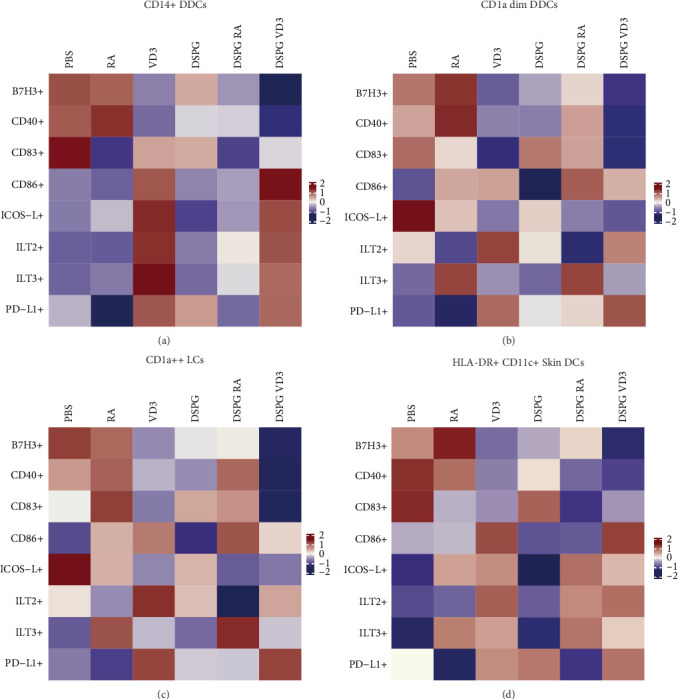
RA and VD3 liposome treatment modulate expression of skin DC-activating and tolerogenic surface markers. Frequencies of marker+ cells were evaluated using spectral flow cytometry and analysis in FlowJo. The resulting dataset was uploaded to Tercen, which scaled the data using the *R* package scale function and generated heatmaps. (A) Heatmap indicating scaled frequencies of CD14+ DDCs, (B) CD1a dim DDCs, (C) CD1a++ LCs, or (D) total HLA-DR+ CD11c+ skin DCs expressing different DC activation or inhibitory markers. *N* = 5.

Expression of LC activation markers was largely unaltered, while we observed a trend for RA-mediated ILT3 induction and VD3-liposome-mediated PD-L1 induction, as well as, B7H3 reduction (Figure 4A–F, Supporting Information 5: Figure S5A–F). Thus, VD3 and RA treatment of LCs also pointed towards tolerogenic modulation, albeit less significantly than in the other subsets. Expression of all measured DC markers is shown in a heatmap per skin DC subset and in total HLA-DR+ CD11c+ skin DCs (Figure 5A–D). Taken together, VD3 liposome injection reduces activation and induces tolerogenic marker expression of CD14+ and CD1a dim DDCs, comparably to soluble vitamin, while the immunoregulatory effects of RA and RA liposomes are most pronounced in CD1a dim DDCs (Figure 5).

**Figure 6:**
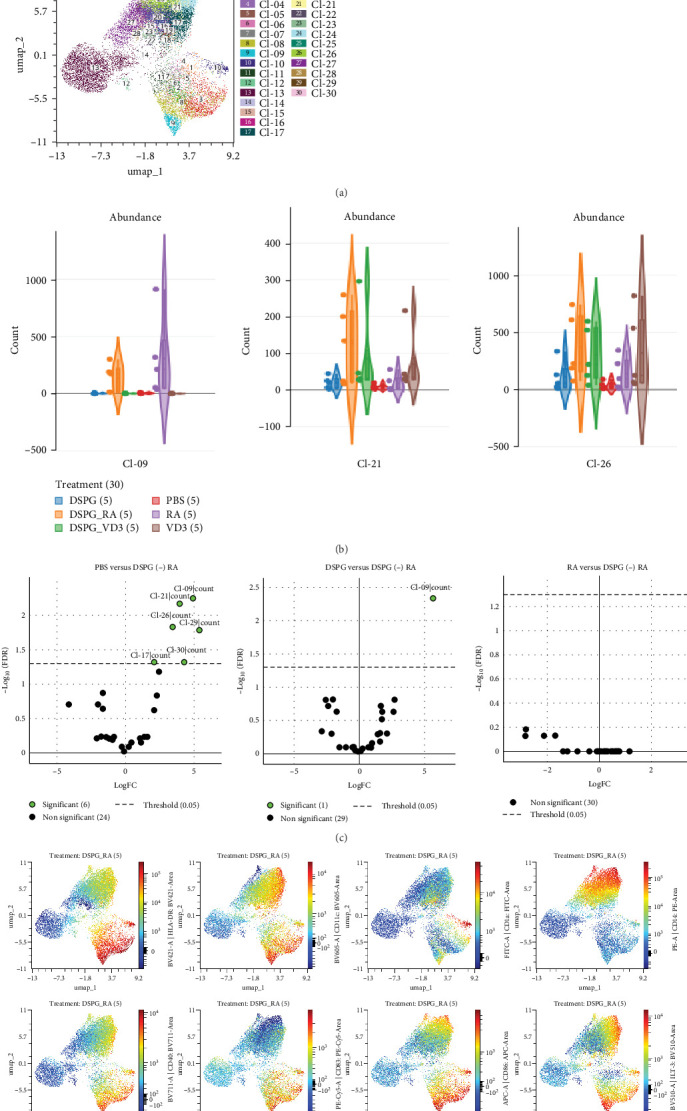
Unbiased analysis of RA-liposome-treated skin DCs reveals differentially expressed clusters. Spectral cytometry data were loaded in the OMIQ platform and UMAP dimensionality reduction, as well as, FlowSom clustering using 30 clusters was carried out. Cluster abundances were compared using the EdgeR algorithm. (A) Overlay dotplots indicating the 30 clusters on UMAP analysis resulting from FlowSom clustering. (B) Violin plots showing frequencies (counts per total of cells) in each cluster that showed significant differences between treatment conditions. (C) Volcano plots after EdgeR analysis indicating differential expression of clusters between PBS and DSPG RA, DSPG and DSPG RA or RA and DSPG RA. (D) UMAP visualization of skin DCs treated with DSPG RA, showing expression of all measured markers. *N* = 5.

**Figure 7:**
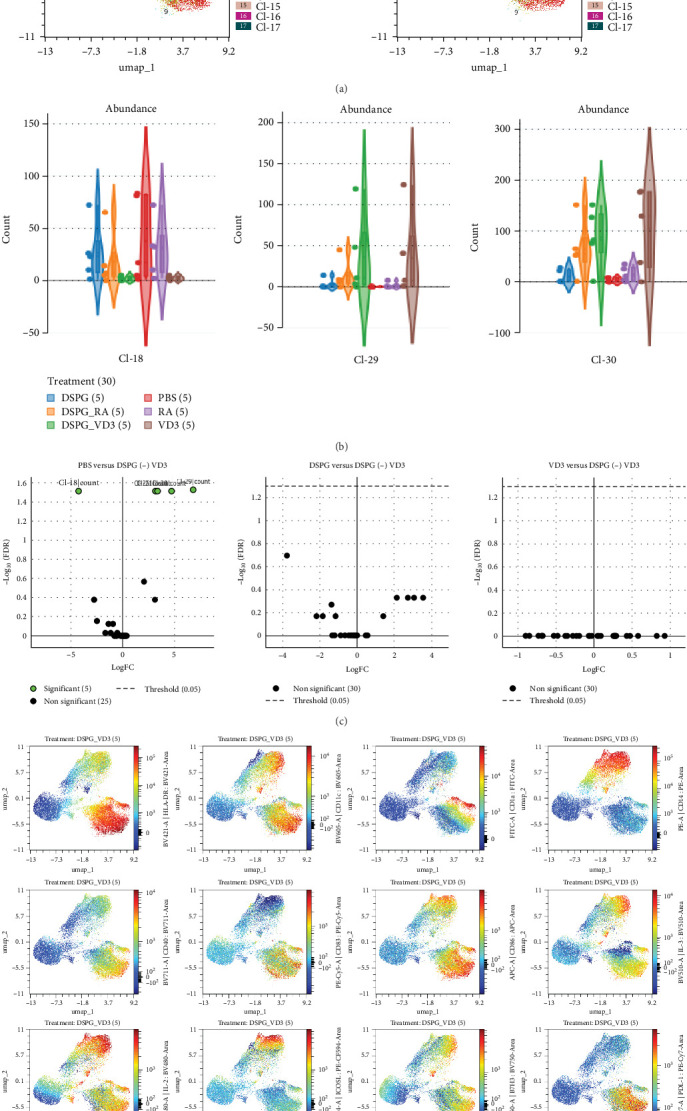
Unbiased analysis of VD3-liposome-treated skin DCs reveals differentially expressed clusters. Spectral cytometry data were loaded in the OMIQ platform and UMAP dimensionality reduction, as well as, FlowSom clustering using 30 clusters was carried out. Cluster abundances were compared using the EdgeR algorithm. (A) Overlay dotplots indicating the 30 clusters on UMAP analysis resulting from FlowSom clustering. (B) Violin plots showing frequencies (counts per total of cells) in each cluster that showed significant differences between treatment conditions. (C) Volcano plots after EdgeR analysis indicating differential expression of clusters between PBS and DSPG VD3, DSPG and DSPG VD3 or VD3 and DSPG VD3. (D) UMAP visualization of skin DCs treated with DSPG VD3, showing expression of all measured markers. *N* = 5.

Complementing above targeted analysis we carried out UMAP dimensionality reduction and unsupervised FlowSom clustering of live, singlet skin crawl-outs. This analysis identified 30 clusters, out of which cluster 9 was exclusively present in DSPG RA and soluble RA treatment conditions (Figure 6A–C). Cluster 9 encompassed activated skin DCs which were HLA-DR++ CD11c+, CD1a dim, with high expression of CD40, CD83, CD86, and tolerogenic ILT-3, PD-L1, and B7H3 (Figure 6D). Furthermore, compared to PBS, soluble and liposomal RA, but also VD3, induced clusters 21 and 26 (Figure 6B). Both vitamin treatments led to differential expression of cluster 30, which was CD11c+ and CD14++ with dim HLA-DR, featuring limited expression of activation markers and high expression of ILT2 and ICOS-L (Figure 7A–D), aligning with our observations on CD14+ DDC induction. Furthermore, the minor cluster 29, also expressing high CD14, was increased in abundance upon both vitamin treatments. As in the targeted analysis, we observed no significant differences in clusters or their abundance between soluble and liposomal vitamin-treated DCs (Figure 6C,Figure 7C). Hence, our unsupervised results align with manual analysis of CD1a dim and CD14+ DDCs.

### 2.3. Soluble and Liposome-Vitamin-Treated Skin DCs Induce Development of T Cells With Regulatory Phenotype

**Figure 8:**
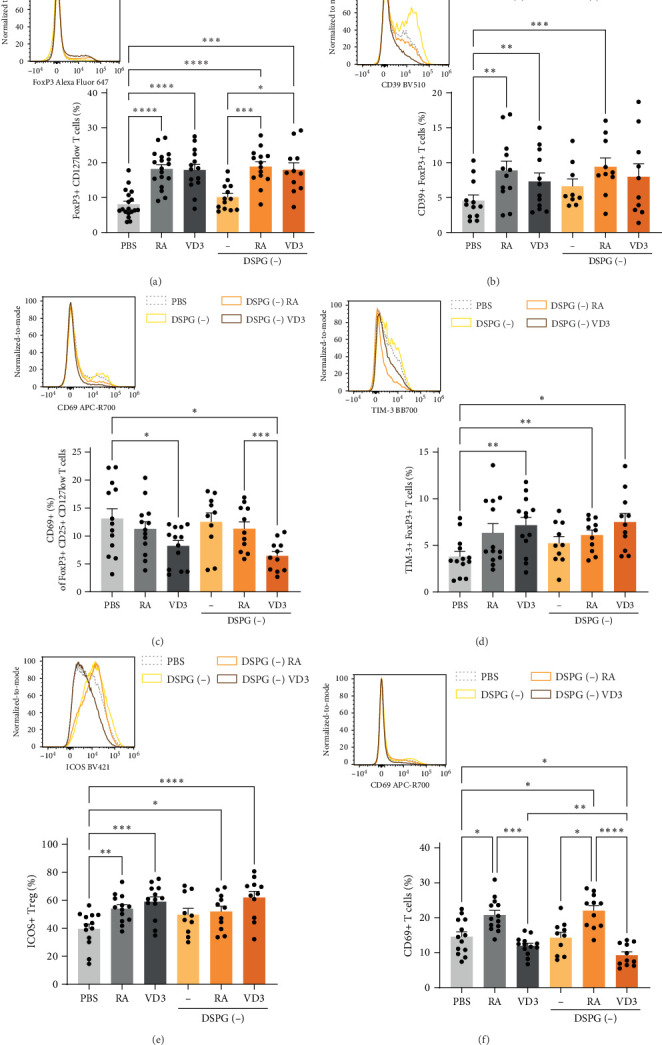
RA- or VD3-liposome-treated skin DCs induce FoxP3+ CD127low and ICOS+ Tregs expressing functional Treg markers. Allogeneic naïve CD4+ T cells were cocultured with RA- or VD3-liposome-exposed irradiated skin crawl-out cells in the presence of 10 pg/mL SEB. FoxP3+ CD127low CD4+ T cells were analyzed for functional Treg marker expression using spectral flow cytometry. (A) Example histogram of FoxP3-expressing single CD4+ T cells and frequencies of FoxP3+ CD127low CD4+ T cells per injection condition are shown. (B) Example histogram of CD39-expressing FoxP3+ CD127low T cells and frequencies of CD4+ T cells coexpressing CD39 and FoxP3 are shown. (C) Example histogram of CD69-expressing FoxP3+ CD127low T cells and frequencies of CD69+ cells within FoxP3+CD127low T cells are shown. (D) Example histogram of TIM-3-expressing FoxP3+ T cells and frequencies of CD4+ T cells coexpressing TIM-3 and FoxP3 are shown. (E) Example histograms of ICOS-expressing single CD4+ T cells and frequencies of CTLA-4+ ICOS+ FoxP3+ T cells (ICOS+ Treg) are shown. (F) Example histogram of CD69-expressing single CD4+ T cells and frequencies of CD69+ total CD4+ T cells. *N* = 10–13. Error bars depict mean ± SEM. Statistical significance was calculated using mixed-effects analysis with Tukey's correction for multiple comparisons. *⁣*^*∗*^*p* ≤ 0.05. *⁣*^*∗∗*^*p* ≤ 0.01. *⁣*^*∗∗∗*^*p* ≤ 0.001. *⁣*^*∗∗∗∗*^*p* ≤ 0.0001. Colors on histograms represent: grey dashed line-PBS, yellow thin line-DSPG, orange line-DSPG RA, brown line-DSPG VD3. Individual data points: number of donors tested per condition (independent experiments).

As both RA and VD3 liposomes modulate the expression of inflammatory and tolerogenic features of skin DCs with reduced expression of DC activation markers and induced expression of ILT3 or PD-L1 (Figure 5D, Supporting Information 6: Figure S6A–F), we examined the effect of vitamin-liposome-treated skin DCs on the development of naïve CD4+ T cells. We cocultured RA- and VD3-primed skin crawl-out cells, containing high amounts of HLA-DR + CD11c+ skin DCs (Figure S1C) with allogeneic naïve CD4+ T cells and analyzed T cell phenotype after 10–12 days by spectral flow cytometry (Figure S7A–C). Both RA- and VD3-liposome-treated DCs enhanced FoxP3+ CD127low T cells similarly to soluble-vitamin-treated DCs (Figure 8A). Close to 100% of FoxP3+ T cells were CD127 low (Figure S7C, representative donor).

Next, we determined the coexpression of Treg markers in combination with FoxP3 on T cells induced by differentially treated skin DCs (Figure 8B–D, Supporting Information 8: Figure S8A–G). Of note, RA- and RA-liposome-treated DCs and VD3-treated DCs induced T cells coexpressing FoxP3 and the immunosuppressive ecto-enzyme CD39 [34], suggesting enhanced suppressive capacity of these T cells (Figure 8B). Contrasting this observation, within FoxP3+ CD127low CD25+ cells (46.5%–90% of FoxP3+CD127low T cells, Supporting Information 8: Figure S8A) we noted a reduction in frequencies of CD69+ cells upon coculture with VD3-treated skin DCs, indicating a potentially reduced suppressive capacity or reduced activation of induced FoxP3+ cells (Figure 8C) [35]. Underlining their tolerogenic phenotype, we observed an enhanced percentage of T cells coexpressing the immune checkpoint receptor for Galectin-9, TIM-3 [36] and FoxP3 upon treatment with RA and VD3-liposome-primed DCs compared to PBS injection (Figure 8D). ICOS+ Tregs, identified as a distinct Treg subset with enhanced suppressive capacity [37] were also induced upon coculture with RA and VD3-liposome-treated DCs compared to PBS (Figure 8E). In the total population of CD4+ T cells, RA- but not VD3-liposome-treated DCs induced frequencies of CD69+ cells (Figure 8F) [34]. T cells primed by RA- or VD3-liposome-treated skin DCs showed a trend of suppressive function towards CFSE-labeled CD4+ memory T cells, especially when using the division index for analysis, indicating functional Treg activity (Figure S9A,B). Thus, both RA-liposome- and VD3-liposome-treated skin DCs lead to the induction of known T regulatory phenotypes confirming that injections of RA and VD3 promote the migration of tolerogenic skin DCs from ex vivo skin explants.

### 2.4. Skin DCs Treated With RA or VD3 Liposomes Do Not Affect Tr1 Tregs but Modulate T Cell Polarization

**Figure 9:**
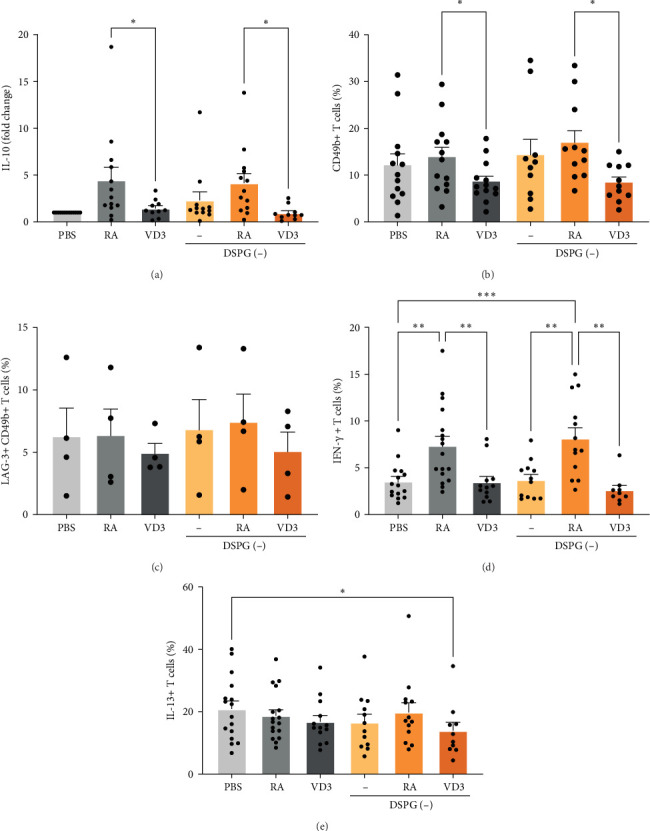
RA- or VD3-liposome-treated skin DCs do not induce Tr1 cells but modulate T cell polarization. Allogeneic naïve CD4+ T cells were cocultured with RA- or VD3-liposome-exposed irradiated skin crawl-out cells in the presence of 10 pg/mL SEB for 10–12 days. IL-10 expression was evaluated in culture supernatants using sandwich ELISA. Expression of CD49b, LAG-3, IFN-γ, and IL-13 were evaluated with flow cytometry. (A) IL-10 production by aCD3/28-restimulated CD4+ T cells is shown in different crawl-out coculture conditions, normalized to the control PBS condition (fold change). *N* = 8–13. (B) T cell expression of CD49b in resting T cells, *n* = 10–13, or (C) T cell expression of CD49b together with LAG-3 in aCD3/28-restimulated T cells is shown of *n* = 3–4. (D-E) Frequencies of IFN-γ+ (Th1) cells and IL-13+ (Th2) cells are shown. *N* = 8–16. Error bars indicate mean ± SEM. Statistical significance was calculated using mixed-effects analysis with Tukey's or Šidák's correction for multiple comparisons. *⁣*^*∗*^*p* ≤ 0.05. *⁣*^*∗∗*^*p* ≤ 0.01. *⁣*^*∗∗∗*^*p* ≤ 0.001. Individual data points: number of donors tested per condition (independent experiments).

Type 1 regulatory (Tr1) Tregs are characterized by high IL-10 production and coexpression of CD49b and LAG-3 [38]. To investigate Tr1 induction by RA-or VD3-liposome-treated crawl-out DCs, we measured IL-10 secretion, as well as, CD49b and LAG-3 surface expression after aCD3/28 restimulation of cocultured CD4+ T cells (Figure S10A,B). Interestingly, both RA- and RA-liposome-treated DCs induced expression of IL-10 by T cells (Figure 9A, Supporting Information 10: Figure S10A). However, neither RA- nor RA-liposome-treated DCs induced expression of CD49b on resting T cells or Tr1 cells, compared to PBS or empty liposomes (Figure 9B,C).

Next, we investigated expression of IFN-γ and IL-13 as key cytokines to assess T helper cell polarization towards Th1 and Th2 cells. Interestingly, skin DCs induced high levels of IL-13+ T cells and low levels of IFN-γ+ T cells, indicating a Th2 bias of skin DC-primed T cells (Figure S10C, Figure 9D,E) [33, 39]. While VD3- and VD3-liposome-treated DCs did not modulate Th1 or Th2 development, coculturing soluble-RA- and liposome-RA-treated skin DCs with naïve T cells induced Th1 cells compared to the PBS control and compared to VD3 treatment (Figure 9D). We did not observe modulation of Th2 outgrowth (Figure 9E). Hence, our data suggest that RA and VD3-treated skin DCs do not induce Tr1 type Tregs. In fact, RA-treated skin DCs promote development of Th1 cells.

## 3. Discussion

The skin is an ideal tissue for the application of vaccines. Therefore, we investigated the effect of intradermal injection of liposomes carrying tolerogenic adjuvants RA or VD3 on primary DC subsets in human skin. Our data suggest that intradermal application of RA or VD3 in soluble or liposomal form enhances CD14+ DDC migration, and the expression of coinhibitory molecules on CD1a dim DDCs, promoting the development of regulatory T cells. These data serve as encouraging precedents for a future in vivo study utilizing dermal delivery.

As DCs have the capacity to reprogram the immune system towards a state of tolerance, there is a growing interest in developing DC-targeting therapies for the treatment of autoimmunity or allergy [8, 40]. Due to easy accessibility, the skin is an attractive site for reaching primary DCs with immune modulating compounds. In contrast to subcutaneous administration, the intradermal route allows direct targeting of the extensive network of heterogeneous skin DCs which are key in initiating a diverse range of T cell responses [41–46].

Here, we investigated the effect of intradermal injection of RA or VD3 liposomes on human primary skin DCs. Incorporating RA or VD3 in liposomes did not alter their capacity to promote tolerogenic responses via skin injection. Nevertheless, encapsulation of RA or VD3 into liposomes allows a combination with specific disease-related antigens. In addition, the use of nanoparticles enables targeting to specific DC subsets by incorporating DC-specific ligands [27, 40]. We employed RA- and VD3-loaded liposomes to probe the therapeutic concept of in vivo DC modulation. Liposomes have the added benefit of shielding therapeutic compounds carried in them. Unlike soluble adjuvants, liposomes may be more durable upon injection resulting in enhanced availability of the carried component to APCs in the skin, as well as transdermal entry into the circulation with systemic immune modulation as a consequence [47].

We show that intradermal application of RA in both soluble and liposomal forms increased the number of migratory CD14+ DDCs, accompanied by a reduction in migratory CD1a dim DDCs, which was comparable to VD3 and VD3 liposome injection. Such a shift in migratory skin DC populations may convey therapeutic benefits in treating chronic inflammatory conditions as CD14+ DDCs have previously been described as tolerogenic, while CD1a dim DDCs were associated with pro-inflammatory effects [41, 43, 48]. A possible alternative explanation to the CD14+ DDC migration enhancing effect of the vitamins, which we cannot exclude, is that other skin DC subsets increase CD14 expression upon treatment. This has been described in several in vitro studies demonstrating enhanced CD14 expression on DCs by VD3 treatment, with tolerogenic T cell priming consequences [11, 49]. Nevertheless, we observe tolerogenic effects of migratory DCs after skin vitamin injection. Compared to CD1a dim DDCs, the CD14+ DDC population expressed lower levels of DC costimulatory molecules CD40, CD83, and CD86, confirming observations in previous studies and serving as an explanation for intrinsic tolerogenic effects of this skin DC subset [32, 50].

RA liposomes induced the coinhibitory molecule ILT3 in CD1a dim DDCs in contrast to CD14+ DDCs. Hence, tolerogenic effects of RA injection may stem from simultaneous stark induction of CD14+ DDCs together with tolerogenic modulation of the more inflammatory CD1a dim DDC subset. These modulations were confirmed by unbiased analysis, demonstrating the emergence of new skin DC clusters characterized by an activated population expressing ILT-3 upon RA treatment, and lesser activated CD14+ cells expressing ILT2 and PD-L1.

In total skin DCs, treatment with DSPG RA liposomes led to reduced expression of DC activation markers as well as induced expression of coinhibitory ILT3. Interestingly, these DSPG RA-treated DCs induced allogeneic T cells expressing FoxP3, as well as, the separate subset of ICOS+ Tregs, suggesting tolerogenic modulation of the T cell compartment. Furthermore, the induced FoxP3+ T cells frequently coexpressed the ecto-enzyme CD39, which depletes extracellular ATP and thereby, functions as a Treg marker [51]. Similar observations have been made in vivo in mice, where injection of DSPG RA liposomes stimulated the generation of Tregs [52]. We selected anionic DSPG liposomes for this study, as these empty carriers have been demonstrated to induce murine FoxP3+ Tregs in vivo [29]. Interestingly, empty DSPG liposomes also induced CD14+ DDC migration compared to PBS, as well as, reducing CD40 and inducing PD-L1 on CD14+ and CD1a dim DDCs. However, these observations did not translate into increased induction of FoxP3+ T cells. Hence, DSPG liposomes may convey an added benefit to injecting soluble adjuvants due to their stimulatory capacity of DC migration, but this remains to be investigated in an (antigen-specific) in vivo setting in the near future.

Besides inducing FoxP3+ Tregs under the influence of VD3 [32], CD14+ DDCs also have been characterized as IL-10 producers that can induce Tregs [41, 46, 53]. IL-10 stimulates the development of Tr1s and is also highly produced by these T cells [38, 54]. Despite a trend of enhanced IL-10 production by CD4+ T cells that were induced by RA- or DSPG RA-primed DCs, we did not observe a significant induction of Tr1-type cells in our study. Thus, other Treg subsets may be the source of increased IL-10. Besides Tr1s, ICOS+ Tregs are also characterized as IL-10 producers, serving as one likely explanation of the increase in IL-10 production in RA- or RA-liposome-skin-DC-primed T cells [55]. The increased IL-10 production induced by RA-treated skin DCs contrasts with earlier observations where mucosal-DC-derived RA inhibited the induction of IL-10 production by naïve CD4+ T cells [56]. In this study, RA-primed skin DCs rather stimulated the outgrowth of FoxP3+ T cells. However, previous data from our group align with current observations, namely, that RA-primed DCs stimulate both the outgrowth of FoxP3+ T cells and IL-10 producers [57].

The effects of RA on T cell polarization are still controversial. Besides RA-DC-mediated induction of FoxP3+, IL-10+ and CCR9+ gut-homing T cells [14, 56, 57], direct treatment of T cells with RA or via monocytes was linked to Th2 induction, but also to stabilization of Th1 cells [58–61]. Interestingly, ICOS has also been described in balancing Th1, Th2 cell subsets, and several studies argue that ICOS is linked to Th2 cell differentiation [62–64]. We observed a significant induction in IFN-γ+ Th1 cells, but no modulation of IL-13+ Th2 cell subsets when using RA-primed skin DCs. This observation suggests that RA liposomes may be used in allergen immunotherapy, where an induction of allergen specific Th1 cells alongside Tregs conveys therapeutic benefits [65, 66].

Aligning with our previously published results, soluble VD3 and VD3 liposomes led to enhanced migration of CD14+ DDCs [32, 33]. In CD14+DDCs only VD3 treatment reduced activation markers and induced ILT3, underlining additional tolerogenic modulation of this subset. Within the more activated CD1a dim DDCs VD3 induced coinhibitory PD-L1 indicating tolerogenic reprogramming of the more inflammatory CD1a dim DDC subset, as induction of DC ILT3 and PD-L1 have both been described to lead to T cell anergy or Treg induction [19, 67, 68]. Indeed, ILT3 induction by RA or VD3 has been observed previously in moDCs [33, 67, 69].

In the current study, VD3-liposome-treated skin DCs induced FoxP3+ T cells in coculture, aligning with results of our previous study utilizing soluble VD3 for intradermal injection, as well as, with in vivo findings in a rat model of encephalomyelitis [32, 70]. Additionally to FoxP3 induction, VD3-liposome-treated skin DCs also induced ICOS+ Tregs and FoxP3+ T cells coexpressing CD39. Thus, treatment of skin DCs with VD3 liposomes promotes their capacity to induce tolerogenic T cell development, supporting further use of this adjuvant in an in vivo vaccine platform.

VD3-treated skin DCs did not induce IL-10 producing T cells, nor LAG-3+ CD49b+ T cells, indicating no induction of Tr1 type Tregs in coculture [38]. Moreover, VD3-liposome-treated skin DCs also did not modulate T helper cell polarization, in spite of our previous studies pointing out a Th1 inhibitory function of VD3-treated DCs [33, 49, 71]. Lack of induction of IL-10 producing T cells and inhibition of Th1 cells could be due to reduced expression of ICOS-L and B7H3 in VD3-treated skin DCs. ICOS-L and B7H3 are key coinhibitory molecules involved in induction of IL-10 producing T cells and inhibition of Th1 cells, respectively [72, 73]. However, VD3-modulated clinical grade ex vivo DCs also boast low ICOS-L expression and do not express B7H3, which suggests that VD3 injection in our study induces a tolerogenic DC phenotype despite the counterintuitive reduced expression of these two coinhibitory markers [11, 74]. VD3-DC-stimulated T cells may also be influenced by other tolerogenic molecules, such as TGF-β, which is a major driver of FoxP3+ Tregs while suppressing IL-10-producing Tr1 cells [75, 76]. Another recently identified regulatory molecule derived from CD14+ CD141+ DDCs and VD3-moDCs is the neuropeptide urocortin 2, which induces Tregs and dampens T cell inflammation in the skin [77]. The exact mechanism behind induction of FoxP3+ T cells by skin DCs in our study will need to be explored further in the future.

In conclusion, we show that liposome-loaded and soluble RA or VD3 are effective tolerogenic immunomodulators for the induction of tolerance in situ, in primary human skin DCs. Taken together, our findings support further development of a nanoparticle platform using RA and VD3 to induce tolerance via in vivo intradermal DC vaccination.

## 4. Materials and Methods

### 4.1. Liposome Preparation and Quality Control

Liposomes were manufactured at the Leiden Academic Center for Drug Research using the thin film dehydration-rehydration method, as described previously [29, 31, 33]. For the anionic DSPG formulation, 1,2-dioleoyl-sn-glycero-3-phosphocholine (DOPC) (Tm = −15°C), the charged lipid DSPG (Tm = 54.4°C), and cholesterol were dissolved in chloroform and mixed in a molar ratio of 4:1:2 DOPC:DSPG:cholesterol. To obtain RA- or VD3-loaded liposomes, 450 μg RA (Sigma-Aldrich, St Louis, Missouri, USA) dissolved in chloroform or 150 μg VD3 (Santa Cruz, Dallas, Texas, USA) dissolved in ethanol was added to approximately 3 mg of lipids. Measurement of vitamin content, lipid content, and quality control of liposomes was performed as described elsewhere [31, 33]. All formulations had a homogenous size of less than 200 nm, with ζ-potential corresponding to expected electrical charge. The formulations were stable for 3 months (Tables 1 and 2).

### 4.2. Intradermal Injections and Culturing of Human Skin Explants

Abdominal or breast human skin was obtained from Almere hospital (Flevoziekenhuis, Almere, The Netherlands) after cosmetic surgery. The skin was stored at 4°C and used within 24 h after surgery. Intradermal injections were performed with 50 μL of PBS, RA (30 μM), VD3 (25 μM), and both empty and vitamin-loaded DSPG liposomes. Liposomes and soluble compounds were diluted in PBS. Lipid concentration of liposomes injected was adjusted to the concentration of vitamin injection and ranged from 400 to 2500 μg/mL dependent on loading efficiency of vitamin compounds per liposome batch. Lipid concentration of injected empty liposomes was the average of lipid concentrations used for RA liposome and VD3 liposome injection. Intradermal injections were applied using insulin needles (0.6 mm × 25 mm Microlance; BD Biosciences). Immediately after injection, a skin biopsy of 6 mm in diameter was taken with a sterile biopsy punch (Kai Medical) from the ex vivo human skin with the injection site centrally located. Subsequently, the subcutaneous fat was removed. For each treatment, 3–24 biopsies were harvested. Biopsies were temporarily placed for approximately 1–2 h in 0.5 mL IMDM containing 1% FCS with the epidermal side up, after which the biopsies were transferred into 1 mL of IMDM supplemented with 10% FCS and 100 ng/mL GM-CSF and cultured at 37°C for 3 days. After removal of the biopsies, crawl-out cells were placed in 4°C, harvested and pooled per treatment condition. The percentage and counts of specific subsets of total migrated HLA-DR+ CD11+ DCs, CD14+ DDCs, CD1a dim DDCs and CD1a++ LCs were analyzed using spectral flow cytometry. Skin DC frequencies, or activation and tolerogenic marker expression were not assessed in conditions with skin DC counts below 400, or DC subset counts below 100. Crawl-out cells were stained in PBA (0.5% BSA, 0.05% Natrium-Azide) supplemented with 1% HS (Lonza) and 2% FCS (FACS buffer) with the markers listed in Table 3. Surface expression was measured on a SP6800 Sony Spectral Analyzer (Sony Biotechnology) or a FACS Canto II (BD). FlowJo software (Tree Star, Ashland, OR) was used for data analysis. Heatmaps were generated with Tercen using data output from FlowJo. Data were scaled in Tercen with the “scale” function of base *R*, which first centered the data by subtracting the mean frequency of each marker (row means) from the frequency in corresponding injection conditions (columns) and then dividing the resulting number by the standard deviation of each row.

### 4.3. Stimulation of Naïve CD4+ Cells by Crawl-Out DCs and T Cell Phenotypical Analysis

Naïve CD4+ T cells were isolated as described previously [33]. Crawl-out skin cells were γ-irradiated (30 Gy) prior to T cell stimulation to prevent any possible proliferation of contaminating skin T cells. After irradiation the crawl-out cells were washed three times in IMDM 10% FCS medium. To determine the stimulatory capacity of migratory DCs, 20.000 γ-irradiated crawl-out cells were cocultured with 20.000 allogenic naive CD4+ T cells in 200 μL IMDM 10% FCS in 96-well flat-bottom plates in the presence of superantigen Staphylococcal enterotoxin B (SEB, 10 pg/mL; Toxin Technology). At day 4 or 5, the cell suspension of the coculture was transferred to fresh medium supplemented with 10 U/mL IL-2 (Novartis, Basel, Switzerland) in a 24-well flat-bottom plate. IL-2-supplemented medium was refreshed every other day until day 10–12 when staining was performed using the flow cytometry panel assembled in Table 4.

### 4.4. T Cell Suppressor Assay

300.000 CD4+ naive T cells were induced by coculture with 120.000 γ-irradiated crawl-out DCs in IMDM 10% FCS and collected after 5 days of incubation. Subsequently, the CD4+ T cells (test cells) were extensively washed in IMDM 1% FCS, counted and irradiated (30 Gy) to prevent expansion. Next, CD4+ memory T cells (target cells) from the same donor as test cells were stained with CarboxyFluorescein Succinimidyl Ester (CFSE) for cell proliferation measurements. 50.000 test cells were cocultured with 25.000 target cells, 1500 TNF-α, IL-1β, and LPS-matured moDCs in IMDM 10% FCS, in triplicate. After 5 days, the proliferation of the target T cells was determined by flow cytometry on a FACS Canto II (Becton Dickinson). Additionally, the division index was extracted using the FlowJo proliferation modelling tool, reflecting the average number of cell divisions each cell has undergone.

### 4.5. Analysis of T Cell-derived Cytokine Production

At day 10 or 11 of coculture, resting CD4+ T cells were restimulated with 10 ng/mL phorbol-12-myristate 13-acetate (PMA) (Sigma-Aldrich), 1 μg/mL ionomycin (Sigma-Aldrich), and 10 μg/mL brefeldin A (Sigma-Aldrich) in IMDM 10% FCS. T cells were fixed with 3.7% formaldehyde in PBS, permeabilized with saponin PBA, and stained at room temperature with anti-IFN-γ-FITC (clone 25723.11, BD Biosciences) and anti-IL-13-PE (clone JES10-5A2, BD Biosciences). Flow cytometry was performed on a FACS Canto II (BD). Alternatively, a total of 100.000 resting T cells in triplicate per condition were restimulated for 24 h with 0.5 μg/mL anti-CD3 and 1 μg/mL anti-CD28 (Sanquin Research). Cells were analyzed for Tr1 markers with spectral flow cytometry using anti-CD49b and anti-Lag-3-PE-Dazzle (clone 11C3C65) staining. IL-10 was measured in supernatants via ELISA [71].

### 4.6. Statistics and Unbiased Data Analysis With Dimensionality Reduction

Flow-cytometric analyses were performed using FlowJo software (Ashland, OR, USA, version 10.7.1 for Windows). Graphs were plotted with GraphPad Prism version 9.3.1 (GraphPad, La Jolla, CA). As datasets contained missing data, mixed-effects analyses with Tukey's or Šidák's correction for multiple comparisons were performed. A value of *p* ≤ 0.05 was considered significant.

For unbiased analysis, skin DC data of five donors and T cell data of 10 donors were transferred to the OMIQ data analysis platform (Omiq, Inc, Santa Clara, CA, USA). Within OMIQ, data were Arcsinh cofactor 400 transformed, followed by gating based on side and forward scatter morphology, and subsequent live cell (where applicable) and singlets gating. For skin DCs downsampling to 5000 cells per sample and for T cells downsampling to 30.000 cells per sample, or 5000 FoxP3+CD127 low CD25+ cells followed. Dimensionality reduction was carried out using the PCA, opt-SNE, and UMAP algorithms, applying measured skin DC or T cell markers as features. Simultaneously, automated clustering was carried out using the same features, with FlowSOM. Cluster abundances between treatments were assessed using cluster percentages and cluster counts of the total single-cell or FoxP3+ CD127 low CD25+ populations. Significance testing comparing cluster abundances between two treatment conditions each was done using the edgeR algorithm, embedded in OMIQ.

## Figures and Tables

**Table 1:** Physicochemical properties of RA and VD3-loaded liposome formulations.

Liposome formulation	Lipid composition	Mean *Z*-average diameter (nm)	± SD	PdI	± SD	Mean *ζ*-potential (mV)	± SD	LE (%)	± SD
DSPG (−) RA	DOPC:DSPG:CHOL	159	8.5	0.14	0.02	−41.0	4.3	60	16.3
DSPG (−) VD3	DOPC:DSPG:CHOL	165	4.0	0.14	0.01	−36.2	16.2	56	8.1

*Note:* Characteristics are shown as mean ± SD of *n* = 3 different batches. LE, loading efficiency of RA or VD3.

**Table 2:** Stability measurements of DSPG RA or VD3-loaded liposome formulations.

DSPG (−) RA						
Time (months)	*Z*-ave (nm)	± SD	PdI	± SD	*ζ*-Potential (mV)	± SD
0	159	8.53	0.14	0.02	−41.05	4.26
1	151	11.15	0.16	0.01	−48.31	3.99
2	163	7.67	0.14	0.04	−47.68	14.32
3	166	12.89	0.18	0.02	−45.16	8.44

**DSPG (−) VD3**						
**Time (months)**	***Z*-ave (nm)**	**± SD**	**PdI**	**± SD**	***ζ*-Potential (mV)**	**± SD**

0	165	3.96	0.15	0.01	−36.18	16.17
1	176	2.01	0.15	0.02	−44.96	2.89
2	184	4.41	0.15	0.03	−40.13	4.31
3	189	23.23	0.25	0.12	−53.35	0.97

*Note:* Stability was monitored over the course of 3 months using *Z*-ave (mean *Z*-average diameter), PdI and average *ζ*-potential measurements. Measurements are expressed as mean ± SD of *n* = 3 liposome batches.

**Table 3:** Spectral flow cytometry panel for skin DC phenotyping.

Marker	Antibody clone	Fluorochrome	Supplier
HLA-DR	L243	BV421	BD Pharmingen
ILT2	GHI/75	BV480	BD Biosciences
ILT3	ZM3.8	BV510	BD Biosciences
CD11c	B-ly6	BV605	BD Biosciences
CD40	5C3	BV711	BD Horizon
B7H3	7-517	BV750	BD Biosciences
CD86	2331 (FUN1)	APC	BD Pharmingen
CD14	MΦP9	PE	BD Pharmingen
ICOS-L	2D3	PE-CF594	BD Biosciences
CD83	HB15e	PE-Cy5	BD Pharmingen
PD-L1	M1H1	PE-Cy7	eBioscience
CD1a	HI149	FITC	BD Pharmingen
Viability dye	—	APC-ef780	eBioscience

**Table 4:** Spectral flow cytometry panel for T cell phenotyping.

Marker	Antibody clone	Fluorochrome	Supplier
ICOS	D10.G4.1	BV421	BioLegend
TIGIT	741182	BV480	BD Biosciences
CD39	TU66	BV510	BD Biosciences
CD49b	12F1	BV605	BD Biosciences
PD-1	EH12.1	BB515	BD Biosciences
TIM-3	344823	BB700	BD Biosciences
CD127	h-IL-7R-M2	PE	BD Biosciences
CTLA-4	BNI3	PE-Cy5	BD Pharmingen
CD25	M-A251	PE-Cy7	BioLegend
CD69	FN50	APC-R700	BD Biosciences
FoxP3	259D	AF647	BioLegend

## Data Availability

The data that support the findings of this study are available from the corresponding author upon reasonable request.

## Supporting Information

Supporting Information 1 Figure S1. Gating strategy of skin DCs, skin DC subsets, and DC activation or tolerogenic markers within skin DCs and skin DC subsets. (A) Crawl-out cells were gated based on forward and side scatter. After gating for live cells and single cells, HLA-DR+ CD11c+ skin DCs were gated as shown. The skin DC population was further gated based on CD1a and CD14 expression into the three skin DC subsets: CD14+ DDCs, CD1a dim DDCs, and CD1a++ LCs. Within total skin DCs and the respective subsets, expression of CD40, CD83, CD86, B7H3, ICOS-L, ILT2, ILT3, and PD-L1 was determined as shown. (B) Frequencies of CD14+ DDCs expressing CD141 are shown for one donor. (C) Frequencies of CD1a dim DDCs expressing CD141 are shown for one donor. (D) Frequencies of live HLA-DR+ CD11c+ crawl-out DCs are shown per injection condition. *N* = 8. (E) Frequencies of HLA-DR+ CD11c+ skin DCs among crawl-out cells. *N* = 16–17. Error bars indicate mean ±SEM. Statistical significance was calculated using mixed-effects analysis with Tukey's correction for multiple comparisons. Individual data points: number of donors tested per condition (independent experiments).

Supporting Information 2 Figure S2. RA and VD3 liposome injection modulate migration of skin DC subsets from skin explants. Counts of migrating skin DC subsets or frequencies of subsets within HLA-DR+ CD11c+ skin DCs were evaluated in the different injection conditions using flow cytometry. (A) Counts of different DC subsets migrating from ex vivo skin biopsies per injection condition. (B–D) Frequencies of CD14+ DDCs, CD1a dim DDCs, or CD1a++ LCs within HLA-DR+ CD11c+ skin DCs are shown. *N* = 18–19. Error bars indicate mean ± SEM. Statistical significance was calculated using mixed-effects analysis with Tukey's correction for multiple comparisons. *⁣*^*∗*^*p* ≤ 0.05. *⁣*^*∗∗*^*p* ≤ 0.01. *⁣*^*∗∗∗*^*p* ≤ 0.001. *⁣*^*∗∗∗∗*^*p* ≤ 0.0001. Individual data points: number of donors tested per condition (independent experiments).

Supporting Information 3 Figure S3. RA and VD3 liposome treatment modulate expression of skin DC-activating and tolerogenic markers in CD14+ DDCs. MFI of markers was evaluated in the different injection conditions within HLA-DR+ CD11c+ skin DCs, which were CD14+, using spectral flow cytometry. Conditions with counts less than 100 CD14+ DDCs were excluded from analysis. (A,B) Example histograms of one donor and MFI of CD40, and CD86 in CD14+ DDCs is shown. (C–E) Example histograms of one donor and MFI of ILT2, ILT3, and B7H3 is shown within CD14+ DDCs. *N* = 8–10. Error bars indicate mean ± SEM. Statistical significance was calculated using mixed-effects analysis with Tukey's correction for multiple comparisons. *⁣*^*∗*^*p* ≤ 0.05. *⁣*^*∗∗*^*p* ≤ 0.01. *⁣*^*∗∗∗*^*p* ≤ 0.001. Colors on histograms represent: grey dashed line-PBS, yellow thin line-DSPG, orange line-DSPG RA, brown line-DSPG VD3. Individual data points: number of donors tested per condition (independent experiments).

Supporting Information 4 Figure S4. RA and VD3 liposome injection reduce expression of activation markers and induce expression of tolerogenic markers in CD1a dim DDCs. MFI of markers was evaluated in the different injection conditions within HLA-DR+ CD11c+ skin DCs, which were CD1a dim, using spectral flow cytometry. Conditions with counts less than 100 CD1a dim DDCs were excluded from analysis. (A,B) Example histograms of one donor and MFI of CD40, and CD83 in CD1a dim DDCs is shown. (C–F) Example histograms of one donor and MFI of ILT3, PD-L1, B7H3, and ICOS-L is shown within CD1a dim DDCs. *N* = 7–10. Error bars indicate mean ± SEM. Statistical significance was calculated using mixed-effects analysis with Tukey's correction for multiple comparisons. *⁣*^*∗*^*p* ≤ 0.05. *⁣*^*∗∗*^*p* ≤ 0.01. *⁣*^*∗∗∗*^*p* ≤ 0.001. *⁣*^*∗∗∗∗*^*p* ≤ 0.0001. Colors on histograms represent: grey dashed line-PBS, yellow thin line-DSPG, orange line-DSPG RA, brown line-DSPG VD3. Individual data points: number of donors tested per condition (independent experiments).

Supporting Information 5 Figure S5. RA and VD3 liposome injection do not alter expression of activation markers on LCs while RA liposome injection shows a trend for inducing ILT3 and VD3 liposome injection induces PD-L1 and reduces B7H3 expression. MFI of markers were evaluated in the different injection conditions within HLA-DR+ CD11c+ skin DCs, which were CD1a++, using spectral flow cytometry. Conditions with counts less than 100 CD1a++ LCs were excluded from analysis. (A–C) Example histograms of one donor and MFI of CD40, CD83, and CD86 in CD1a++ LCs is shown. (D–F) Example histograms of one donor and MFI of ILT3, PD-L1, and B7H3 is shown within CD1a++ LCs. *N* = 3–7. Error bars indicate mean ± SEM. Statistical significance was calculated using mixed-effects analysis with Tukey's correction for multiple comparisons. *⁣*^*∗*^*p* ≤ 0.05. *⁣*^*∗∗*^*p* ≤ 0.01. Colors on histograms represent: grey dashed line-PBS, yellow thin line-DSPG, orange line-DSPG RA, brown line-DSPG VD3. Individual data points: number of donors tested per condition (independent experiments).

Supporting Information 6 Figure S6. RA and VD3 liposome treatment modulate expression of skin DC-activating and tolerogenic markers. Frequencies of markers were evaluated in the different injection conditions within HLA-DR+ CD11c+ skin DCs. Conditions with less than 400 skin DCs were excluded from analysis. (A–F) Frequencies of CD40+, CD83+, ILT3+, B7H3+, PD-L1+, and ICOS-L+ skin DCs are shown. *N* = 9–11. Error bars indicate mean ± SEM. Statistical significance was calculated using mixed-effects analysis with Tukey's correction for multiple comparisons. *⁣*^*∗*^*p* ≤ 0.05, *⁣*^*∗∗*^*p* ≤ 0.01. *⁣*^*∗∗∗*^*p* ≤ 0.001. *⁣*^*∗∗∗∗*^*p* ≤ 0.0001. Individual data points: number of donors tested per condition (independent experiments).

Supporting Information 7 Figure S7. Gating strategy for evaluating CD4+ T cell tolerogenic markers in crawl-out-DC-stimulated cocultures and suppressor assay using CD4+ T cells. After 10-12-day coculture with differently primed crawl-outs, CD4+ T cells were stained for Treg subset and functional markers. (A) Frequency of indicated markers and populations were determined from the single-cell gate of T cells. (B) Coexpression of FoxP3 with indicated markers was determined in the single cell population of T cells using quadrant gates. (C) FoxP3+ CD127 low T cells were gated as shown; subsequently, frequencies of the depicted CD25-coexpressing subpopulations were determined within FoxP3+ CD127 low cells.

Supporting Information 8 Figure S8. (A) Frequencies of CD25+ cells within FoxP3+CD127low T cells. *N* = 11–13. (B–G) Frequencies of CD39+, CTLA-4+, CTLA-4+ ICOS+, PD-1+, TIGIT+, and TIM-3+ cells coexpressing CD25, within the FoxP3+ CD127low T cell population. *N* = 10–13. Error bars depict mean ± SEM. Statistical significance was calculated using mixed-effects analysis with Tukey's correction for multiple comparisons. *⁣*^*∗*^*p* ≤ 0.05. *⁣*^*∗∗*^*p* ≤ 0.01.

Supporting Information 9 Figure S9. DSPG-RA- and VD3-treated and VD3 treated skin crawl-outs induce marginally suppressive CD4+ T cells. After 6-day coculture with differently primed crawl-outs CD4+ T cells were cultured with activated moDCs and CFSE-labeled bystander memory T cells for 6 days. Proliferation of memory T cells was measured with flow cytometry. To obtain the division index, the proliferation modelling tool in FlowJo was used. (A) Proliferation of and (B) division index of memory T cells in different treatment conditions. *N* = 3–4, colors of dots indicate separate donors. Individual data points: number of donors tested per condition (independent experiments).

Supporting Information 10 Figure S10. (A) IL-10 expression in supernatants of T cells stimulated with differently treated skin DCs, and restimulated with aCD3/28 of *n* = 8–13, (B) representative examples for determining frequency of Tr1 cells within single T cells, and (C) or IFN-γ+ and IL-13+ single T cells per injection condition. Individual data points: number of donors tested per condition (independent experiments).
